# Removal by Tracheotomy of a Safety Pin from Larynx after One Year

**Published:** 1890-09

**Authors:** H. L. Fountain

**Affiliations:** Bryan


For Daniel's Texas Medical Journal.

### REMOVAL BY TRACHEOTOMY OF A SAFETY PIN WHICH HAD REMAINED IN THE LARYNX ONE YEAR.

BY H. L. FOUNTAIN, M. D., BRYAN.

A CHILD four years old was brought to me by its mother with thefollowing history: One year previous to her visit, the child, by a sudden inspiration, sucked a medium sized safety pin into the larynx. It was immediately seized with convulsions and difficult breathing; a physician was sent for, but the pin was not removed. The severe symptoms gradually subsided, until the child was fairly comfortable, though there was a complete loss of speech. The mother did not seek further medical advice until the child's throat began to swell, and it had frequent attacks of coughing and difficult breathing, which would terminate in a free discharge of pus through the mouth. These attacks were of daily occurrence. The laryngoscope revealed a very much swollen condition of the glottis,—the rima being almost closed, and the base of the tongue; and by careful manipulation of the mirror I could detect a dark object projecting up between the vocal cords, but on acconnt of the restless and frightened state of the child, it was impossible to seize it with the forceps, but this was fortunate, as I afterward found the pin was open and the point up, and any forcible effort to extract it with the forceps would have certainly resulted in serious injury to the vocal cords. The following day the child was anæsthetized and the trachea opened as high up as possible without injury to the larynx. The pin was immediately brought into view and removed without difficulty; it was very much corroded and covered with pus.

The wound was closed and the child recovered quite rapidly, though there was some suppuration, due no doubt, to the septic condition of the larynx. The power of speech gradually improved and is now as good as that of any child four years old.

This case was not reported because the operation presented any unusual features, but on account of the length of time the pin remained in the larynx, and the complete recovery of speech after its complete loss, I thought a report of the case might interest some of the profession.

